# Case Report: Surgical resection of ovarian teratoma in anti-NMDAR encephalitis complicated by severe pneumonia

**DOI:** 10.3389/fonc.2025.1659143

**Published:** 2025-10-02

**Authors:** Dengrong Zhang, Wangjing Ren, Xin Shao, Yi Fu, Wei Zhang

**Affiliations:** ^1^ Department of Critical Care Medicine, Leshan People’s Hospital, Leshan, China; ^2^ Department of Gynaecology and Obstetrics, Leshan People’s Hospital, Leshan, China; ^3^ Department of Pharmacy, Leshan People’s Hospital, Leshan, China

**Keywords:** autoimmune encephalitis, anti-NMDAR encephalitis, ovarian teratoma, severe pneumonia, surgical removal

## Abstract

Anti-N-methyl-D-aspartate receptor (NMDAR) encephalitis is one of the most recognized forms of autoimmune encephalitis. We report the case of a 25-year-old woman with ovarian teratoma-associated anti-NMDAR encephalitis complicated by severe pneumonia. Despite treatment at multiple hospitals, she remained comatose with involuntary perioral myoclonus and severe pulmonary infection while intubated. After aggressive anti-infective therapy at our center, her condition deteriorated, and extubation failed; subsequent tracheostomy stabilized the infection and allowed uneventful laparoscopic removal of the ovarian teratoma. Postoperatively, consciousness returned, convulsions ceased, and pulmonary infection continued to improve. The patient was discharged in good condition. Early screening for ovarian teratomas is essential. When teratoma-associated anti-NMDAR encephalitis presents with severe pneumonia, aggressive infection control, meticulous airway management, and timely surgical resection combined with immunotherapy can substantially improve clinical outcomes.

## Introduction

Anti-N-methyl-D-aspartate receptor (NMDAR) encephalitis is a form of autoimmune encephalitis that has received substantial attention in recent years, with an incidence of 0.6 per 100,000. The condition was first described by Vitaliani et al. in 2005 (1), and its association with ovarian teratomas was later identified by Dalmau (2). Anti-NMDAR encephalitis predominantly affects women and has a reported mortality rate of up to 7% (3). Current evidence suggests that neural tissue within teratomas produces anti-NMDAR antibodies, which cross the blood–brain barrier and trigger inflammation in the central nervous system (4, 5). Most patients have a preceding upper respiratory tract infection, and the disease usually presents with impaired consciousness and motor deficits (6), both of which increase susceptibility to pulmonary infection. Progression to severe pneumonia may be life-threatening. When ovarian teratoma-associated anti-NMDAR encephalitis is diagnosed, meticulous airway management and prevention of severe pneumonia are essential. Timely surgical intervention can reduce the risk of complications such as multiple organ dysfunction syndrome, refractory status epilepticus, and sepsis (7, 8), thus improving clinical outcomes. Written informed consent was obtained from the patient for publication of this information, in accordance with the Declaration of Helsinki.

## Case report

In May 2023, a 23-year-old patient was diagnosed with bilateral ovarian teratomas by transvaginal ultrasound during evaluation for irregular menstrual periods. On December 5, 2023, after an upper respiratory tract infection, she developed sudden-onset abnormal mental behavior and received treatment at a hospital in Zhejiang for 1 week without improvement. She was then transferred to the Emergency Intensive Care Unit of West China Hospital in Sichuan.

During hospitalization, the patient was comatose and exhibited limb convulsions and perioral involuntary movements. An *Enterobacter cloacae* complex–induced pulmonary infection was diagnosed. Because of progressive hypoxemia, she was intubated and empirically treated with piperacillin-tazobactam. Cerebrospinal fluid antibody testing confirmed autoimmune encephalitis (anti-NMDAR antibody titer: 1:100). Despite administration of high-dose methylprednisolone (1 g daily for 4 days) and intravenous immunoglobulin (20 g daily for 4 days), the patient continued to exhibit limb convulsions and perioral involuntary movements. A multidisciplinary team concluded that the teratoma was likely contributing to autoimmune encephalitis. Surgical resection of the tumor combined with anti-infective therapy was recommended as a means of potentially alleviating her convulsive symptoms. After comprehensive discussions with her family regarding surgical risks, prognosis, treatment costs, and expected duration, the family requested her discharge. She was subsequently admitted to the intensive care unit of our hospital on December 31, 2023.

**Figure 1:**
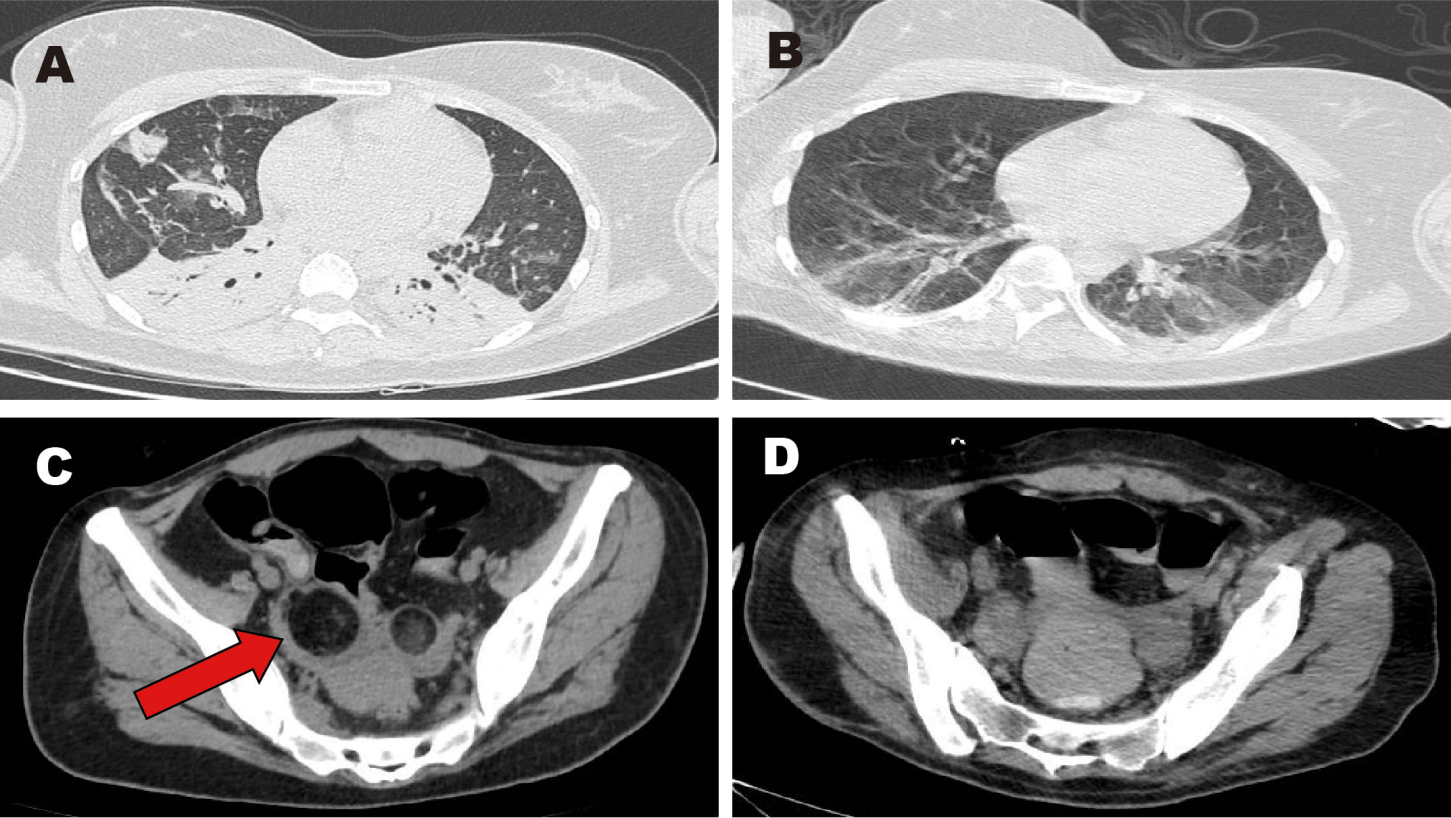
Images of chest and pelvic computed tomography (CT). **(A, C)** CT scans obtained before surgery. **(B, D)** CT scans obtained after surgery. Red arrow: mixed-density lesion in the right adnexa.

Upon admission to our hospital, she was intubated and sedated for pain management. Piperacillin-tazobactam (4.5 g every 8 hours for 4 days) was initially administered to manage the pulmonary infection (**Figure 1A**: inflammation in both lower lungs with partial atelectasis). On days 1–4, bronchoalveolar lavage fluid analysis identified carbapenem-resistant *Acinetobacter baumannii*. Based on antimicrobial susceptibility testing, the antibiotic regimen was adjusted to cefoperazone-sulbactam (3 g every 8 hours for 1 month). However, the patient continued to experience recurrent fevers, with temperatures fluctuating above 38°C; she expectorated a large volume of yellow purulent sputum. Repeated laboratory tests indicated worsening infection markers and deteriorating arterial blood gas parameters. On days 1–8, colistimethate sodium (500,000 units every 12 hours for 19 days) was added to the antimicrobial regimen. Due to prolonged intubation, severe pulmonary infection, excessive oral secretions, and a weak cough reflex, percutaneous tracheostomy was performed on day 9. Anti-infective therapy continued concurrently. During hospitalization, serial bronchoalveolar lavage fluid tests showed a gradual decline in *A. baumannii*, as well as detection of small amounts of *Stenotrophomonas maltophilia* and *Pseudomonas aeruginosa*. Infection markers (**Table 1**) and chest computed tomography (CT) findings improved; the patient exhibited only occasional low-grade fever. On days 1–27, tigecycline (50 mg every 12 hours for 8 days) was administered as monotherapy. Body temperature and laboratory indices then normalized. Subsequently, an antibiotic de-escalation strategy was implemented using cefoperazone-sulbactam (2 g every 8 hours for 2 days).

**Table 1:** Laboratory test results.

Indicator/Date (normal range)	12.31	1.3	1.7	1.11	1.18	1.25	2.2	2.6
Total white blood cell count (3.5–4.5×10^9^)	15.69	9.54	9.90	12.15	19.51	12.04	8.82	7.88
Neutrophil percentage (40%–75%)	84.5	76.6	64.4	74.2	81.6	78.6	68.5	67.1
Lymphocyte percentage (20%–50%)	9.7	17.2	30	17.9	12.8	14.1	18.9	21.7
High-sensitivity C-reactive protein (0–6 mg/L)	6.97	–	3.95	61.49	6.43	3.37	2.67	3.77
Procalcitonin (≤0.05 ng/mL)	0.2	0.21	0.2	0.66	0.16	0.21	<0.1	<0.1
Interleukin-6 (<7 pg/mL)	12.6	22.22	3.95	34.75	25.64	14.32	10.19	8.70
Arterial blood pH (7.35–7.45)	7.381	7.392	7.460	–	–	7.423	7.425	–
Arterial oxygen partial pressure (80–105 mmHg)	78	114	87	–	–	131	171	–
Arterial carbon dioxide partial pressure (35–45 mmHg)	39.2	41.7	37.9	–	–	37	39.4	–
Arterial oxygen saturation (95%–98%)	95	98	97	–	–	100	100	–

**Figure 2:**
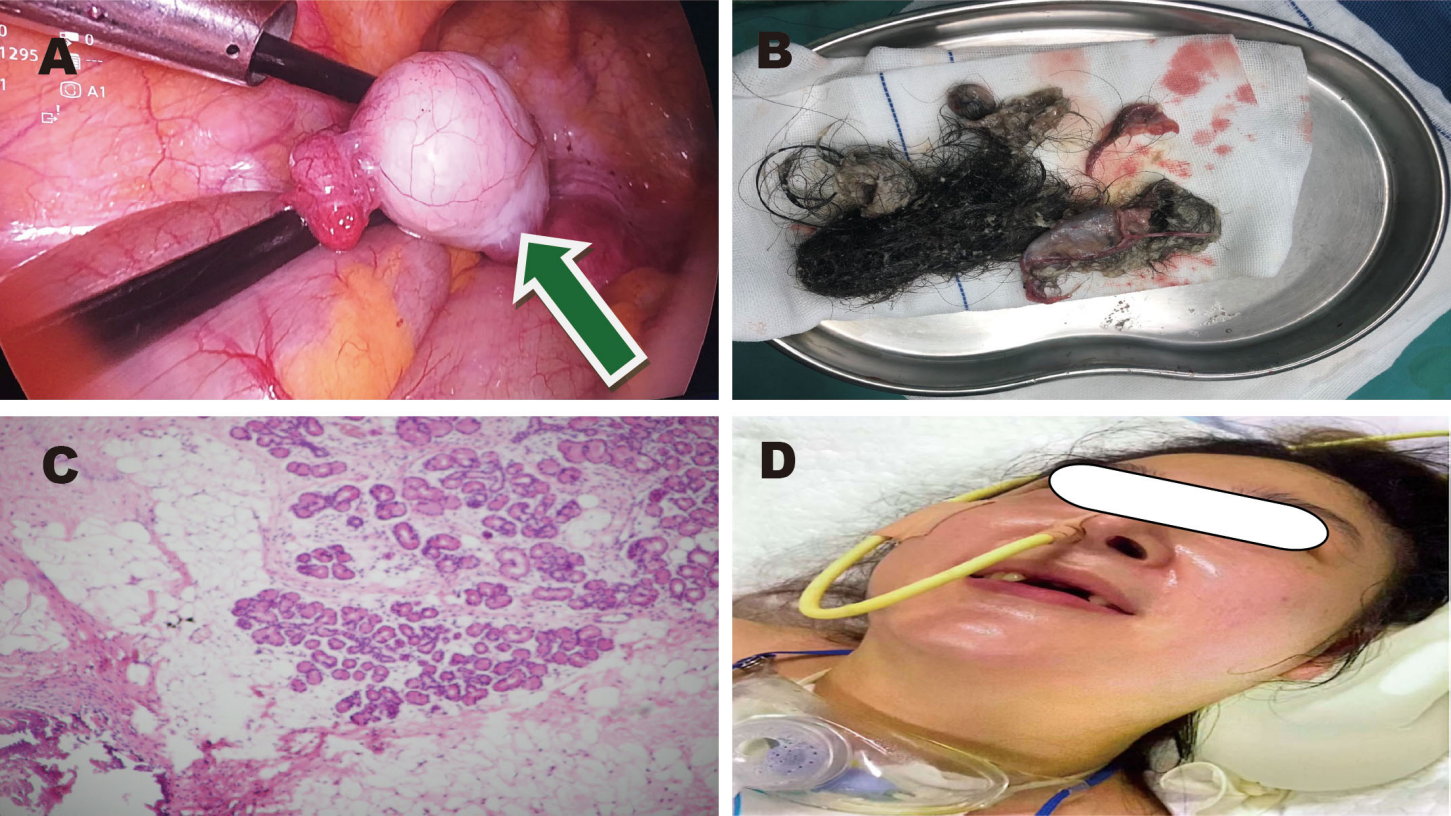
Images of ovarian teratoma. **(A)** Green arrow: laparoscopic view of the teratoma. **(B)** Postoperative specimen. **(C)** Hematoxylin and eosin staining. **(D)** Clinical condition of the patient.

The patient’s main manifestations of epilepsy included impaired consciousness and recurrent convulsions involving the lips, face, and limbs. Glucocorticoids were administered in combination with multiple antiepileptic and sedative agents, including levetiracetam (particularly effective for partial seizures and primary generalized tonic-clonic seizures), valproate (effective for generalized and partial seizures), phenobarbital (suitable for status epilepticus), lacosamide (primarily used for monotherapy or adjunctive treatment of partial-onset seizures), midazolam, and dexmedetomidine. However, seizure control remained inadequate. EEG: Severely abnormal, with continuous, diffuse, high- to very-high-amplitude delta activity predominating across all leads and interspersed with a moderate amount of beta activity. Abdominal CT showed mixed-density lesions in both adnexal regions, measuring approximately 5.0×4.8 cm on the right and 2.3×2.3 cm on the left, suggestive of teratomas (**Figure 1C**). After multidisciplinary consultation, the ovarian teratoma was identified as the likely trigger of autoimmune encephalitis. On January 25, 2024, the patient underwent laparoscopic bilateral ovarian teratoma resection under general anesthesia (**Figure 2A**). Intraoperative findings included teratomatous tissue containing fat and hair (**Figure 2B**). Postoperative hematoxylin and eosin staining confirmed the histopathological features of teratoma (**Figure 2C**). Postoperative abdominal CT (**Figure 1D**) confirmed the absence of teratomas, and chest CT (**Figure 1B**) showed significant resolution of inflammatory changes in both lower lung lobes. The postoperative EEG remained severely abnormal but showed clear improvement compared with the preoperative tracing. It displayed continuous diffuse moderate- to high-amplitude delta activity, scattered theta waves, and prominent beta activity. The frequency and severity of limb and facial convulsions were also reduced.

On February 2, the patient regained consciousness; she could voluntarily move her limbs and blink on command. On February 6, she was transferred to a local hospital for further rehabilitation. Her tracheostomy was closed on February 12, and she was discharged on February 16. Post-discharge follow-up is being conducted via telephone. Two months postoperatively, the patient communicates normally, performs self-care, and demonstrates normal cognitive function. She is not receiving hormone therapy, and no signs of abnormal mental behavior or recurrence have been observed. Although the patient has a good prognosis, she does not recall experiencing any prodromal symptoms prior to disease onset. Follow-up remains ongoing. A summary of the clinical course is presented in **Table 2**.

**Table 2:** Treatment course.

Days after admission	Symptoms	Main investigations	Treatment
1–4	Frequent abnormal mental behavior, sedation	Lumbar puncture and head CT: No abnormalities. Chest CT: Inflammation in both lower lungs with partial atelectasis. Doppler ultrasound of limbs: Thrombosis in the basilic vein of the right upper arm and cephalic vein.	Intubation with mechanical ventilation; piperacillin-tazobactam combined with cefoperazone-sulbactam for anti-infection; ambroxol for sputum clearance; albuterol sulfate combined with budesonide via nebulization; low molecular weight heparin calcium for thromboprophylaxis; levetiracetam, phenobarbital, lacosamide, and valproate for seizure control; and methylprednisolone sodium succinate for immunosuppression.
5–9	Frequent abnormal mental behavior, sedation, diarrhea	Chest CT: Inflammation in both lower lungs with partial atelectasis and slight increase in bilateral pulmonary inflammation. Stool culture: gram-negative cocci 5%, gram-positive cocci 95%.	Extubation followed by percutaneous tracheostomy; cefoperazone-sulbactam combined with colistimethate sodium for anti-infection; smectite powder and loperamide for diarrhea; and *Bifidobacterium* for gut microbiota regulation.
10–18	Frequent abnormal mental behavior, sedation	Head CT: No abnormalities. Chest CT: Reduced inflammation in both lungs. Abdominal CT: suggestive of teratomas. Liver function tests: alanine aminotransferase 380 U/L, aspartate aminotransferase 150 U/L.	Tracheostomy with humidified oxygen therapy; cefoperazone-sulbactam combined with colistimethate sodium for anti-infection; multidisciplinary consultation and planning for laparoscopic bilateral ovarian teratoma resection; and magnesium isoglycyrrhizinate for hepatic protection.
19–25	Slight improvement in abnormal mental behavior, intermittent sedation	White blood cell count, 12.04×10^9^/L; high-sensitivity C-reactive protein, 3.37 mg/L; procalcitonin, 0.21 ng/mL; interleukin-6, 14.32 pg/mL	Tracheostomy with humidified oxygen therapy; cefoperazone-sulbactam combined with colistimethate sodium for anti-infection; laparoscopic bilateral ovarian teratoma resection performed under general anesthesia on January 25, 2024; and prednisone acetate for immunosuppression.
26–34	Somnolence, occasional facial twitching	Chest CT: Decreased inflammation in both lungs with re-expansion of the lower lobes. Abdominal CT: Postoperative appearance of bilateral adnexal regions.	Tracheostomy with humidified oxygen therapy; and tigecycline combined with cefoperazone-sulbactam for anti-infection.
35–37	Somnolence, follows commands to move limbs, no convulsions	White blood cell count, 7.88×10^9^/L; high-sensitivity C-reactive protein, 3.77 mg/L; procalcitonin, <1 ng/mL; interleukin-6, 8.70 pg/mL	Cefoperazone-sulbactam for anti-infection; prednisone acetate for immunosuppression; transfer to a lower-level hospital on day 37 for further rehabilitation.

## Discussion

In our case, the patient presented with abnormal mental behavior and impaired consciousness, displayed a cerebrospinal fluid anti-NMDAR antibody titer of 1:100, demonstrated diffuse abnormal delta waves on electroencephalography, and exhibited an ovarian teratoma on abdominal CT. These findings met the diagnostic criteria for anti-NMDAR encephalitis (9).

Miao (10) demonstrated that impaired consciousness and motor abnormalities are independent risk factors for pneumonia among hospitalized patients with anti-NMDAR encephalitis. Pneumonia can prolong hospitalization and adversely affect prognosis. The incidence of pneumonia among patients with impaired consciousness is high (76.79%) (11). Patients with a Glasgow Coma Scale score ≤8 exhibit diminished cough reflexes, which increases the risk of aspiration (12). Oromandibular movement disorders are characteristic motor abnormalities in anti-NMDAR encephalitis. These disorders interfere with normal swallowing mechanisms (13), thereby increasing aspiration risk. On admission, the patient was comatose and already intubated. Before transfer, she had developed pneumonia caused by the *Enterobacter cloacae* complex, exhibited persistent perioral myoclonus, and produced copious oral secretions. Although piperacillin–tazobactam was immediately initiated, fever recurred, inflammatory markers rose, and the oxygenation index remained poor. Limb strength and cough reflex were considerably diminished. Respiratory cultures subsequently yielded carbapenem-resistant *A. baumannii*, prompting escalation to cefoperazone–sulbactam plus colistin; nevertheless, pulmonary infection continued to deteriorate. On hospital day 9, while intensifying antimicrobial therapy, we performed a percutaneous tracheostomy. This approach improved sputum clearance, reduced pulmonary sequestration of secretions, and maintained airway patency—interventions that appeared to slow the progression of inflammation. After tracheostomy, inflammatory indices declined and oxygenation improved, although low-grade fever persisted. Ongoing perioral myoclonus and copious oral secretions intermittently obstructed the airway and posed a continued aspiration risk. Given the confirmed diagnosis of anti-NMDAR encephalitis and abdominal CT evidence of bilateral ovarian teratomas, we suspected that resection of the teratomas could attenuate seizure activity and facilitate better control of pulmonary infection. On hospital day 25, after multidisciplinary discussion, laparoscopic bilateral ovarian teratoma resection was performed uneventfully. Postoperatively, the patient regained consciousness, and perioral and limb myoclonus substantially diminished. Inflammatory markers normalized, chest CT demonstrated marked resolution of pulmonary infiltrates, and oxygen saturation remained stable on humidified oxygen without fever.

When an ovarian teratoma is identified in association with anti-NMDAR encephalitis, early surgical resection plus immunotherapy is recommended (3). Failure to promptly remove the teratoma may lead to persistent antigen presentation, resulting in antibody affinity maturation and infiltration of long-lived plasma cells into the bone marrow and brain, ultimately inducing immune tolerance (14). This patient had a confirmed diagnosis of anti-NMDAR encephalitis complicated by severe pneumonia. Management was complex; therefore, early tracheostomy with anti-infective therapy was performed, followed by elective teratoma resection. After surgery, pulmonary infection, seizure activity, and level of consciousness all substantially improved. It remains unclear whether resection within 2 months of symptom onset reduces recurrence risk, given that Erlebach’s meta-analysis did not establish an optimal window (15). In the present case, resection was performed 2 months after onset. To date, the patient has returned to normal life without evidence of relapse.

## Summary

In summary, a high index of suspicion for anti-NMDAR encephalitis is warranted in young female patients presenting with encephalitis and sudden-onset psychiatric symptoms. This disease is characterized by impaired consciousness and motor dysfunction; it carries a high risk of severe pneumonia. When patients deteriorate despite aggressive management of pulmonary infection under endotracheal intubation and extubation cannot be performed, prompt tracheostomy should be considered to slow the progression of lung inflammation. In ovarian-teratoma–associated anti-NMDAR encephalitis complicated by severe pneumonia, securing the airway facilitates subsequent teratoma resection. Removal of the antigenic source improves consciousness and suppresses involuntary movements, thereby enhancing infection control and overall clinical outcomes.

## Data Availability

The original contributions presented in the study are included in the article/supplementary material. Further inquiries can be directed to the corresponding author.
